# The Burden of Survivorship on Hematological Patients—Long-Term Analysis of Toxicities after Total Body Irradiation and Allogeneic Stem Cell Transplantation

**DOI:** 10.3390/cancers13225640

**Published:** 2021-11-11

**Authors:** Michael Oertel, Jonas Martel, Jan-Henrik Mikesch, Sergiu Scobioala, Christian Reicherts, Kai Kröger, Georg Lenz, Matthias Stelljes, Hans Theodor Eich

**Affiliations:** 1 Department of Radiation Oncology, University Hospital Muenster, 48149 Munster, Germany; j_mart18@uni-muenster.de (J.M.); sergiu.scobioala@ukmuenster.de (S.S.); Kai.Kroeger@ukmuenster.de (K.K.); hans.eich@ukmuenster.de (H.T.E.); 2 Department of Medicine A—Hematology, Hemostaseology, Oncology, Pulmonology, University Hospital Muenster, 48149 Munster, Germany; jan-henrik.mikesch@ukmuenster.de (J.-H.M.); christian.reicherts@ukmuenster.de (C.R.); georg.lenz@ukmuenster.de (G.L.); matthias.stelljes@ukmuenster.de (M.S.)

**Keywords:** survivorship, TBI, long-term toxicity, leukemia

## Abstract

**Simple Summary:**

Total body irradiation is an essential large-field technique enabling myeloablation before allogeneic stem cell transplantation. With its field encompassing all organs, a diverse spectrum of toxicities may arise. This work analyzes long-term pulmonary, cardiac, ocular, neurological and renal toxicities in a monocentric patient cohort and identifies possible risk factors. Both the number of patients and the duration of the follow-up period exceed those of many comparable studies in the literature.

**Abstract:**

Total body irradiation is an effective conditioning modality before autologous or allogeneic stem cell transplantation. With the whole body being the radiation target volume, a diverse spectrum of toxicities has been reported. This fact prompted us to investigate the long-term sequelae of this treatment concept in a large patient cohort. Overall, 322 patients with acute leukemia or myelodysplastic syndrome with a minimum follow-up of one year were included (the median follow-up in this study was 68 months). Pulmonary, cardiac, ocular, neurological and renal toxicities were observed in 23.9%, 14.0%, 23.6%, 23.9% and 20.2% of all patients, respectively. The majority of these side effects were grades 1 and 2 (64.9–89.2% of all toxicities in the respective categories). The use of 12 Gray total body irradiation resulted in a significant increase in ocular toxicities (*p* = 0.013) and severe mucositis (*p* < 0.001). Renal toxicities were influenced by the age at transplantation (relative risk: 1.06, *p* < 0.001) and disease entity. In summary, total body irradiation triggers a multifaceted, but manageable, toxicity profile. Except for ocular toxicities and mucositis, a 12 Gray regimen did not lead to an increase in long-term side effects.

## 1. Introduction

Allogeneic stem cell transplantation (alloSCT) has an important role in the treatment of acute leukemia, offering curation in both primary and recurrent/refractory situations [1,2]. Total body irradiation (TBI) is used as a conditioning modality to achieve myeloablation and enable subsequent engraftment of donor marrow independently from pharmacodynamics or pharmacokinetics [1,2]. However, as a large-field technique, TBI may be accompanied by a diverse spectrum of toxicities, such as fatigue, loss of appetite, nausea/vomiting, erythema, mucositis, esophagitis, diarrhea, xerostomia and pneumonitis as (sub-)acute side effects. Long-term sequelae include the impairment of the musculoskeletal, endocrinological, renal, cardiopulmonary and ocular systems and veno-occlusive disease (VOD) in the liver, as well as secondary malignancies [1,2,3,4]. Technical and conceptual innovations in radiotherapy (RT) were introduced in previous decades in order to reduce toxicities and, thereby, the burden of (secondary) disease [3,4]. In spite of this significant progress, the long-term toxicity profile of patients undergoing alloSCT with TBI is still to be defined as previous works have been limited by the number of patients studied and the duration of the follow-up period. This may have led to a putative underestimation of toxicity rates and the extent of the spectrum of side effects. Our analysis aims at providing a detailed, long-term evaluation of a large cohort of patients treated at a hematological center. Toxicity rates and gradings were assessed and analyzed to identify risk factors for the respective organ-specific side effects.

## 2. Materials and Methods

### 2.1. Clinical Data

Retrospective analysis was applied to patients with acute leukemia or myelodysplastic syndrome who underwent alloSCT with a conditioning regimen involving TBI at our hematological center between 2001 and 2018 in this monocentric study. The follow-up was scheduled according to the guidelines of the European Leukemia Net and the German Working Group for Stem Cell Transplantation and Cellular Therapies, with a required minimal follow-up of 1 year (the median follow-up was 68 months). Patients showed a high degree of adherence to the scheduled follow-ups with only infrequent dropouts (e.g., due to a patient’s relocation). Patient data regarding demographics and toxicities were collected from the clinical files and the hospital’s information system (Orbis, Dedalus Health Care, Bonn, Germany), which provided toxicity documentation, doctors’ letters and imaging. Additionally, family physicians were contacted in case of insufficient data/follow-up. For the diagnosis of pneumonia, the presence of bacteria/viruses, and/or a response to antibiotic/antiviral treatment was necessary. The grading of toxicities was executed according to the National Cancer Institute’s Common Terminology Criteria for Adverse Events version 5.0 [5] (CTCAE).

### 2.2. Radiation Treatment

TBI has been performed with various radiation machines during the last 20 years. At the beginning of the evaluation period, a cobalt-60 machine (Philips Health Systems, Amsterdam, The Netherlands) with emitted energies of 1.173 megaelectron-volt and 1.332 megaelectron-volt (averaging 1.22 megaelectron-volt) was used. The patient was positioned 3.5 m from the radiation source in a lying position with a 0.5 cm plexiglass plate in front of them. In 2002, we introduced a PRIMUS linear accelerator for TBI (Siemens Healthineers, Erlangen, Germany) and from 2012 on, a TrueBeam linear accelerator (Varian Medical Systems, Pao Alto, CA, USA) was used. TBI from both linear accelerators was performed as follows: Patients were irradiated in four orthogonal lying positions (anterior–posterior, posterior–anterior and two lateral planes) while situated in a specialized bed that was 5.45 m from the linear accelerator. The applied dose rate was 20 cGray (Gy)/min. A beam-spoiler was positioned directly in front of the patient to guarantee beam buildup. In vivo dosimetry was used via semi-conductor probes (PTW, Freiburg, Germany) with eight measurement positions (head, neck, larynx, thorax, mediastinum and abdomen) to ensure homogenous delivery. An additional larynx block was used in the case of >10% local overdosage. For 12 Gy TBI, lead blocks were applied in front of the mediastinum in the lateral treatment positions to limit irradiation of the lungs. To compensate for potential underdosage in that case, additional anterior–posterior and posterior–anterior fields were used for the axillae and the mediastinum.

### 2.3. Statistical Analysis

Statistical analysis was performed with the program SPSS^®^ version 27.0 (IBM^®^, Armonk, NY, USA). The Kaplan–Meier method was used to visualize and analyze time-dependent events and incidence curves. The interval between treatment and the onset of toxicity (cardiac or pulmonary) was described as either cardiac toxicity-free survival (CTFS) or pulmonary toxicity-free survival (PTFS), and was determined as the time between the first day of RT and the detection of the relevant toxicity event. For both toxicity categories, a landmark analysis was carried out to analyze and visualize the occurrence of long-term RT sequelae. Ocular, neurological and renal toxicity and secondary neoplasia were recorded as non-time-dependent, binary variables. Univariate analysis was performed using the Cox-proportional-hazards regression model (backward elimination (likelihood ratio)) for time-dependent variables and binary logistic regression for non-time-dependent variables in order to determine the relative risks in relation to age, sex, disease, TBI dose, previous cranial radiation (regarding ocular toxicity) and chemotherapy (regarding pulmonary toxicity). Variables with a *p*-value < 0.1 were entered into a multivariate analysis (backward elimination (likelihood ratio)). Fisher’s exact test was used to compare the toxicity rates of TBI doses in cross tabulations. A *p*-value below 0.05 was considered to be statistically significant and no adjustment for multiple testing was made.

## 3. Results

Overall, the 322 patients had a median age of 47 years at transplantation (18–74 years) and a median follow-up of 68 months (M) was identified (see Table 1 for details).

The disease entities present were acute myeloid leukemia (AML) in 213 patients (66.1% of the cohort), acute lymphoid leukemia (ALL) in 91 patients (28.3%) and myelodysplastic syndrome (MDS) in 15 patients (4.7%). The conditioning regimens consisted of fludarabine-8 Gy (either alone in the case of 137 patients (42.5% of the cohort), or in combination with melphalan in the case of 93 patients (28.9%)), cyclophosphamide-12 Gy (68 patients (21.1%)), etoposide-12 Gy (9 patients (2.8%)) or other (15 patients (4.7%)). Data on mucositis were available for 291 patients (90.4% of all patients) with an overall mucositis rate of 86.3%, among which 39.4% were mild to moderate (grades 1 and 2). Compared to that, 57.8% of mucositides were labeled grade 3 and 2.8% were labeled grade 4 (see Table 2). There was a significantly higher rate of severe mucositis (grades 3 and 4) in the 12 Gy TBI regimen (53.2% vs. 83.9% of all mucositides for 8 Gy and 12 Gy, respectively, *p* < 0.001). Organ-specific toxicities were found in 23.9%, 14.0%, 23.6% (6.2% cataracts), 23.9% and 20.2% of all patients for pulmonary, cardiac, ocular, neurological and renal toxicities, respectively. Most patients suffered from grade 1 and 2 toxicities, which account for 64.9%, 70.0%, 80.0%, 87.0% and 89.2% of pulmonary, cardiac, ocular, neurological and renal toxicities, respectively. Regarding these sequelae, 12 Gy TBI only resulted in a significant increase in the rate of ocular toxicities (*p* = 0.013; see Table 2). Two patients displayed VOD (0.6% of the cohort), both received 8 Gy TBI and 17 patients (5.3%) had secondary malignancies. The most prevalent specific toxicities were polyneuropathy (19.6%), sicca-syndrome (16.1%), pneumonia (13.4%), cataract (6.2%) and bronchial obstruction (6.2%). Concerning cardiac toxicities, the most common sequelae were heart failure (3.7%), pericardial effusion (2.5%), atrial fibrillation (2.2%), other arrhythmias (1.9%) and valve disease (1.9%). Other distinctive complications include acute respiratory distress syndrome (ARDS (0.6%)) and radiographic leukoencephalopathy (0.6%).

**Figure 1:**
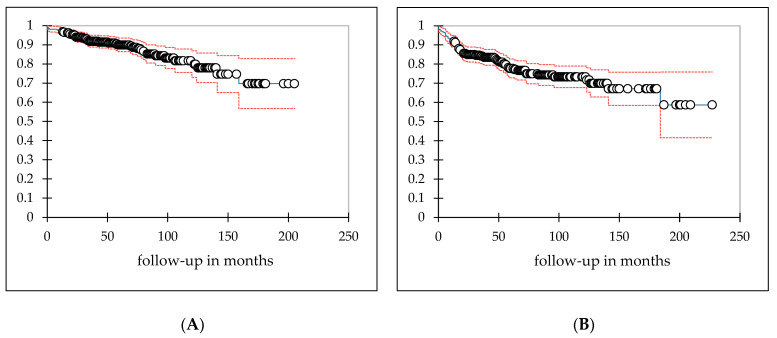
Landmark analysis of patients surviving at least one year after TBI-containing alloSCT. Kaplan–Meier curves for cardiac toxicity-free survival (CTFS; (**A**)) and pulmonary toxicity-free survival (PTFS; (**B**)) are presented above and their respective courses during follow-up, with 95%-confidence intervals, are indicated in red. Mean values were 169.6 months (M; confidence interval: 159.1–180.0 M) for CTFS and 165.6 M (confidence interval: 152.2–178.9 M) for PTFS.

For cardiac toxicity-free survival (CTFS; see Figure 1A), there was no significant association found in the univariate analysis between CTFS duration and the disease entity (ALL vs. AML *p* = 0.371, MDS vs. AML *p* = 0.533), sex (*p* = 0.378) or the TBI dose (*p* = 0.373). Age at the time of transplantation had a significant association with cardiac toxicities in univariate analysis (relative risk (RR): 1.03, confidence interval (CI): 1.00–1.05; *p* = 0.048; see Table 3). The mean CTFS was 169.6 M (CI: 159.1–180.0 M), with toxicity events occurring between 1 and 159 M after radiation was administered.

Regarding pulmonary toxicity-free survival (PTFS; see Figure 1B), there was no significant impact of conditioning chemotherapy (*p* = 0.06), sex (*p* = 0.641), TBI dose (*p* = 0.301), age at transplantation (*p* = 0.139) or disease entity (ALL vs. AML *p* = 0.886, MDS vs. AML *p* = 0.729) on PTFS duration, according to univariate analysis. The mean PTFS was 165.6 M (CI: 152.2–178.9 M), with toxicity events occurring between 1 and 184 M after radiation was administered.

In univariate analysis, renal toxicities were found to be less prevalent for ALL patients (RR: 0.25, CI: 0.11–0.56; *p* = 0.001) and in the 12 Gy conditioning group (RR: 0.29, CI: 0.13–0.67; *p* = 0.004; see Table 3), whereas greater age predisposed patients to a higher level of risk (RR: 1.06, CI: 1.03–1.09; *p* < 0.001). In comparison, sex (*p* = 0.836) had no significant impact. Age at transplantation, disease entity and TBI dose were entered into multivariate analysis, with a significant result found for age at transplantation (RR: 1.06, CI: 1.03–1.09; *p* < 0.001) and ALL vs. AML (RR: 0.31, CI: 0.13–0.74; *p* = 0.008). For neurological toxicities, a univariate analysis did not show a significant association with sex (*p* = 0.089), TBI dose (*p* = 0.436), disease entity (ALL vs. AML *p* = 0.471, MDS vs. AML *p* = 0.713) or age at transplantation (*p* = 0.147). Ocular toxicities were significantly associated with the 12 Gy TBI dose (RR: 2.45, CI: 1.41–4.26; *p* = 0.002) and previous cranial irradiation (RR: 2.12, CI: 1.12–4.00; *p* = 0.020). In contrast, there was no significant association for disease entity (ALL vs. AML *p* = 0.417, MDS vs. AML *p* = 0.320), sex (*p* = 0.285) and age at transplantation (*p* = 0.689). Multivariate analysis showed an association for ocular toxicity with a higher TBI dose (RR: 2.60, CI: 1.49–4.54; *p* = 0.002). Overall, 17 patients suffered from secondary malignancies without significant differences in univariate analysis for TBI dose (*p* = 0.168), sex (*p* = 0.278), disease entity (ALL vs. AML *p* = 0.865, MDS vs. AML *p* = 0.130) or age at transplantation (*p* = 0.078).

## 4. Discussion

Our analysis comprises data from a long-term follow-up of patients who underwent alloSCT after TBI-based conditioning regimens in a large monocentric cohort. Our data reveal a complex, but manageable, toxicity profile spanning different organ systems. Most adverse effects were reported to be mild or moderate. Importantly, there has been no significant association between radiation dose and the side effects for most organs. Greater age at transplantation seems to be a predisposing factor for renal and cardiac sequelae.

Our work demonstrates an average follow-up of 68 M, which exceeds the observation time of most studies from the literature. For example, studies on pulmonary toxicities reveal durations of follow-up between 15 M and 138.8 M; however, most are shorter than 5 years [6,7,8,9,10,11,12,13,14,15,16,17,18,19,20,21,22].

Overall, pulmonary toxicities had a prevalence of 23.9% in the current analysis and, thereby, significantly contribute to morbidity and mortality. In the literature, incidence numbers of pulmonary toxicities (often only registered as pneumonitis) differ between 8.3% and 70.5%, depending on the study cohort and the conditioning regimens utilized [6,7,10,11,12,13,14,15,16,17,19,20,21,22,23,24,25,26]. A comparison of the present study to similar TBI cohorts is provided in Table 4. RT induces a multi-step process of local inflammation resulting in sub-acute pneumonitis and, later, collagen deposition, which causes chronic lung fibrosis [4,27]. Normal tissue-complication probability calculations in a TBI cohort have an estimated rate of 20.3% for overall pneumonitis (both clinical and radiological), 0.6% for clinically symptomatic pneumonitis and 20.4% for lung fibrosis [26]. In contrast, the present analysis only registered infectious pneumonia without accounting for radiation-induced pneumonitis. It should be noted that the diagnosis and subsequent treatment of (subclinical) pneumonites may rely on radiologists and hemato-oncologists, as well as radiation oncologists, having awareness of this ailment. Without regular follow-up imaging, an underestimation of this complication may be assumed. However, the incidence rate of the clinically similar ARDS in our patient cohort was comparable (0.6%) to the calculated rate of clinically manifest pneumonitis, which suggests ambiguity in diagnosis, with one entity perhaps being mistaken for the other. Radiation dose rate is discussed as a significant risk factor, with postulated risk-causing rates varying from 2.5 cGy/min to 41 cGy/min [7,8,9,10,11,12,13,14,15,16,17,23,24,25,28,29,30,31,32]. Cut-off values of 9 cGy/min (autologous) and 15 cGy/min (alloSCT) are identified as a predictor for increased lung toxicity [6,11,15,30], although some evaluations have denied this association [16,32]. Nevertheless, the use of 20 cGy/min in the current analysis did not result in an increased rate of lung toxicity, but instead proved to be feasible and safe when the irradiation time was reduced to a few minutes for each treatment position.

Furthermore, dose rate, along with single-fraction TBI [21,33,34,35], has been postulated as a risk factor for cataract development. A small cohort of 23 patients undergoing alloSCT (the majority of whom were treated with TBI conditioning) showed a cataract rate of 84.6% for single-fraction TBI, in contrast to 0% for a fractionated approach [33]. These results are supprted by the results of two large cohort evaluations in which 5-year cataract incidences of 34% and 61% vs. 10% and 11% were estimated for single-fraction and fractionated TBI, respectively [18,34]. Further risk stratification could be performed by altering the radiation dose rate according to the suggested cut-off values of 4.8 cGy/min and 9 cGy/min [21,34]. Although the dose rate of 20 cGy/min that was used in the present study would fall in the high-risk category for cataract formation, the observed rate of 6.2% was well below the expected value of 54% after 5 years.

Concerning neurological toxicities, an overall incidence rate of 11–59% has been described in the literature, depending on the degree of human leukocyte antigen matching and the risk profile of the hematological disease [36]. Among them, encephalopathies bear significant morbidity and mortality and may be caused by infection or, secondarily, by conditioning therapies such as TBI or busulfan chemotherapy as well as by graft-versus-host disease (GVHD). There were no cases of symptomatic encephalopathy (but 0.6% of patients showed radiological leukoencephalopathy) in the present study’s cohort, which may be due to an underestimation or low-risk profile.

Cardiac side effects were found in 14.0% of patients in our study, with the majority being heart failure, pericardial effusion or arrhythmia. Valvular disease occurring as a toxicity was less common, reflecting the different radiosensitivities of cardiac substructures. The risk of coronary heart disease showed a linear association with the median cardiac dose [37], whereas the risk for valvular disease increased exponentially beyond 30 Gy dose exposure [38]. Comparisons to other TBI cohorts in this regard are difficult to achieve as most do not report cardiac side effects (see Table 4). Previous studies investigating the effect of thoracic radiotherapy on cardiac toxicities did not identify patients’ age at treatment as a consistently significant risk factor [37,38]. However, age may not be the single contributing factor but is likely to be modulated by comorbidities (e.g., diabetes and obesity) or substance abuse (e.g., nicotine and alcohol consumption).

For renal toxicities, the data describing the impact of TBI dose have differed but in our analysis it has not been identified as a significant risk factor. Indeed, Borg et al. described only one case of radiation nephritis in a cohort of 59 patients undergoing 12 Gy-conditioning TBI after 24 M [31]. Mirabell et al. presented details of risk stratification for renal dysfunction after TBI, based on RT dose and GVHD, and showed a high risk for a 13.5 Gy TBI, independent of the presence or absence of GVHD, and 12 Gy TBI with GVHD present [39]. Regarding renal dysfunction, greater age at transplantation is a major risk factor and it may also explain the better outcomes for ALL patients, as these had a lower median age. This is underscored by an analysis by Delgado et al., who defined an age of 30 years or older as a significant risk factor for chronic renal failure (hazard ratio: 6.58) [40].

The rise of modern, intensity-modulated radiation therapy (IMRT) offers the possibility to replace classical TBI approaches (such as those used in the present study) with a more selective technique. IMRT can be used to selectively target bone marrow and/or lymphoid tissue (allowing total marrow irradiation/total lymphoid irradiation) and it can enable dose escalation within these structures while avoiding surrounding at-risk organs [1,3]. Early clinical applications of IMRT-based approaches could confirm a favorable toxicity profile [9,17], but these advantages have to be carefully balanced with a more complex process of decisive contouring, RT planning and execution, as well as quality assurance [1,3]. Furthermore, precise dose coverage and the avoidance of healthy tissue is challenging due to differing patient positioning and patient movement; a multi-institutional study on patient setup accuracy found the largest shifts, from planning computed tomography (CT) to pre-treatment mega-voltage CT, in the lungs, which may result in low-dose variations exceeding 10% [41].

Some of the limitations of the present analysis are due to its monocentric and retrospective character. The documentation of toxicities was sometimes incomplete (e.g., data on mucositides were only available for 90.4% of the patients), which may cause over- or underestimations. Data on comorbidities, toxin exposure and substance abuse were not studied in detail and may reveal additional risk factors. There may also be a substantial study bias due to greater awareness of certain toxicities. Given the lack of detailed documentation (e.g., such as the documentation available on radiological pneumonitis by radiologists and clinicians), further evaluation was not possible. In this context, causal attribution of a given toxicity to chemo- vs. radiotherapy was somewhat ambiguous due to overlapping toxicities caused by radio- and chemotherapy. There has also been a notable, albeit not exclusive, association between pulmonary toxicities and both fludarabine-containing regimens with 8 Gy TBI (97.5% in the 8 Gy cohort) and non-fludarabine-containing regimens with 12 Gy TBI (98.8% in the 12 Gy cohort). Thus, the exact impact of each treatment modality is difficult to decipher and an increase in the 12 Gy group may be masked. Total marrow or total lymphoid irradiations were not included in the study cohort and consequently could not be analyzed. Furthermore, reduced-intensity conditioning was only used in a minority of patients, preventing us from drawing any conclusions about this regimen.

## 5. Conclusions

TBI is a safe conditioning regimen used before alloSCT that results in various, but mostly moderate, toxicities. Except for high-grade mucositis and ocular toxicities, there were no higher rates of side effects in the 12 Gy cohort in comparison to the 8 Gy group. For a reliable toxicity assessment, a sufficient length of follow-up is needed as well as a structured schedule for re-appointments, including clinical and radiological examinations. Radiologists, transplantation physicians and radiation oncologists have to be aware of the type and frequency of these side effects in order to counsel about, monitor and eventually treat these therapy-associated diseases.

## Figures and Tables

**Table 1:** Patient and treatment characteristics are given in absolute numbers and percentages (in parentheses). ALL: acute lymphoid leukemia, AML: acute myeloid leukemia, Gy: Gray, MDS: myelodysplastic syndrome, PLL: prolymphocytic leukemia, TBI: total body irradiation.

Patient Characteristics	*n* (% or Range)
Number of patients	322
Median age at transplantation	47 (18–74)
Sex	
Male	182 (56.5)
Female	140 (43.5)
Diseases	
AML	213 (66.1)
ALL	91 (28.3)
MDS	15 (4.7)
Biphenotypic leukemia	2 (0.6)
T-PLL	1 (0.3)
Graft-versus-host disease	
None	121 (37.6)
Acute only	70 (21.7)
Chronic	128 (39.8)
No information	3 (0.9)
TBI dose	
<8 Gy	4 (1.2)
8 Gy	236 (73.3)
12 Gy	82 (25.5)
Conditioning regimens	
Fludarabine-8 Gy	137 (42.5)
Melphalan-Fludarabine-8 Gy	93 (28.9)
Cyclophosphamide-12 Gy	68 (21.1)
Etoposide-12 Gy	9 (2.8)
Other	15 (4.7)
Previous cranial radiation	
Yes	52 (16.1)
No	267 (82.9)
No information	3 (0.9)

**Table 2:** Overview of toxicities regarding different organ systems and comparison between 8 and 12 Gray conditioning regimens (Fisher’s exact test). Multiple toxicities were possible for each patient. Percentage values of toxicities are given in parentheses and refer to the respective cohort (*n* = 322 for the whole cohort, *n* = 236 for the 8 Gy group and *n* = 82 for the 12 Gy group) except for mucositis. Percentage values of toxicity grades show the distribution of toxicity grades according to Common Terminology Criteria for Adverse Events version 5.0 and add up to 100% in each category. ARDS: acute respiratory distress syndrome, Gy: Gray, TBI: total body irradiation, VOD: veno-occlusive disease.

Toxicity and Grade	*n* * (%)	TBI Dose		*p*-Value
		8 Gy	12 Gy	
VOD	2 (0.6)	2 (0.8)	0	>0.99
Mucositis **	251 (86.3)	188 (85.1)	62 (92.5)	<0.001
Grades 1 and 2	99 (39.4)	88 (46.8)	10 (16.1)	
Grades 3–4	152 (60.6)	100 (53.2)	52 (83.9)	
Pulmonary	77 (23.9)	56 (23.7)	19 (23.2)	0.82
Pneumonia	42 (13.4)	30 (12.2)	11 (11.8)	
Bronchial obstruction	20 (6.2)	14 (5.9)	6 (7.3)	
Dyspnea	9 (2.8)	8 (3.4)	1 (1.2)	
Pleural effusion	7 (2.2)	5 (2.1)	1 (1.2)	
ARDS	2 (0.6)	1 (0.4)	1 (1.2)	
Other	4 (1.2)	4 (1.6)	0	
Grades 1 and 2	50 (64.9)	40 (71.4)	10 (55.5)	
Grades 3–5	27 (35.1)	16 (28.6)	9 (44.5)	
Cardiac	45 (14.0)	31 (13.1)	13 (15.9)	0.58
Heart failure	12 (3.7)	10 (4.0)	1 (1.1)	
Pericardial effusion	8 (2.5)	5 (2.0)	3 (3.3)	
Atrial fibrillation	7 (2.2)	5 (2.0)	2 (2.2)	
Other cardiac arrhythmias	6 (1.9)	3 (1.3)	3 (3.3)	
Valve disease	6 (1.9)	5 (2.0)	1 (1.1)	
Coronary heart disease	5 (1.6)	5 (2.0)	0	
Myocarditis	3 (0.9)	2 (0.8)	1 (1.1)	
Other	4 (1.2)	2 (0.8)	2 (2.2)	
Grades 1 and 2	28 (70.0)	22 (84.6)	6 (46.2)	
Grades 3–5 ***	12 (30.0)	4 (15.4)	7 (53.8)	
Ocular	76 (23.6)	45 (19.1)	30 (36.6)	0.013
Sicca-Syndrome	52 (16.1)	28 (11.4)	23 (24.7)	
Cataract	20 (6.2)	11 (4.5)	9 (9.6)	
Other	13 (4.0)	9 (3.7)	4 (4.3)	
Grades 1 and 2	52 (80.0)	30 (76.9)	21 (84.0)	
Grades 3–5 ****	13 (20.0)	9 (23.1)	4 (16.0)	
Neurological	77 (23.9)	59 (25.0)	17 (20.7)	0.78
Polyneuropathy	63 (19.6)	49 (20.0)	14 (15.0)	
Concentration impairment	15 (6.1)	13 (5.3)	2 (2.2)	
Leukoencephalopathy	2 (0.6)	0	1 (1.1)	
Grades 1 and 2	67 (87.0)	53 (89.8)	14 (82.3)	
Grades 3–5	10 (13.0)	6 (10.2)	3 (17.6)	
Renal	65 (20.2)	57 (24.2)	7 (8.5)	0.002
Grades 1 and 2	58 (89.2)	52 (91.2)	5 (71.4)	
Grades 3–5	7 (10.8)	5 (8.8)	2 (28.6)	
Secondary neoplasia	17 (5.3)	13 (5.5)	4 (3.9)	0.96

* Including conditioning regimens <8 Gray. ** Information on mucositis status was available for 291 patients (90.4% of all patients), percentage values for this category are adjusted relative to this number. *** Toxicity grade not available for 5 patients. **** Toxicity grade not available for 11 patients.

**Table 3:** Univariate and multivariate analysis of different risk factors for organ-specific toxicities.

Toxicity	Variable	Comparison	Univariate Analysis			Multivariate Analysis			Multivariate Analysis		
							Step One			Step Two	
			RR	Range	*p*	RR	Range	*p*	RR	Range	*p*
	Disease	ALL vs. AML	0.71	0.34–1.50	0.371						
		MDS vs. AML	1.46	0.45–4.77	0.533						
Cardiac	TBI dose	12 Gy vs. 8 Gy	0.73	0.36–1.47	0.373						
	Sex	male vs. female	1.32	0.72–2.41	0.378						
	Age at SCT		1.03	1.00–1.05	0.048						
	Disease	ALL vs. AML	1.04	0.63–1.71	0.886						
		MDS vs. AML	0.81	0.25–2.61	0.729						
	TBI dose	12 Gy vs. 8 Gy	0.75	0.44–1.29	0.301						
Pulmonary	Conditioning	flu vs. cy	1.79	0.98–3.30	0.060						
	chemotherapy										
	Sex	male vs. female	0.90	0.57–1.42	0.641						
	Age at SCT		1.01	1.00–1.03	0.139						
	Disease	ALL vs. AML	0.25	0.11–0.56	0.001	0.32	0.13–0.78	0.012	0.31	0.13–0.74	0.008
		MDS vs. AML	1.07	0.33–3.50	0.910	0.99	0.30–3.32	0.991	1.00	0.30–3.34	0.997
Renal	TBI dose	12 Gy vs. 8 Gy	0.29	0.13–0.67	0.004	0.90	0.35–2.33	0.826			
	Sex	male vs. female	0.94	0.55–1.63	0.836						
	Age at SCT		1.06	1.03–1.09	<0.001	1.06	1.03–1.09	<0.001	1.06	1.03–1.09	<0.001
	Disease	ALL vs. AML	1.23	0.70–2.17	0.471						
		MDS vs. AML	1.25	0.38–4.10	0.713						
Neurological	TBI dose	12 Gy vs. 8 Gy	0.94	0.81–1.10	0.436						
	Sex	male vs. female	0.63	0.37–1.07	0.089						
	Age at SCT		1.02	1.00–1.04	0.147						
	Disease	ALL vs. AML	1.27	0.72–2.23	0.417						
		MDS vs. AML	1.77	0.58–5.42	0.320						
	TBI dose	12 Gy vs. 8 Gy	2.45	1.41–4.26	0.002	2.29	1.22–4.30	0.010	2.60	1.49–4.54	0.001
Ocular	Sex	male vs. female	1.33	0.79–2.26	0.285						
	Age at SCT		1.00	0.99–1.02	0.689						
	Previous cranial	yes vs. no	2.12	1.12–4.00	0.020	1.38	0.67–2.85	0.387			
	irradiation										
	Disease	ALL vs. AML	0.90	0.28–2.92	0.865						
Secondary		MDS vs. AML	3.28	0.70–15.33	0.130						
neoplasia	TBI dose	12 Gy vs. 8 Gy	0.44	0.14–1.42	0.168						
	Sex	male vs. female	1.80	0.62–5.21	0.278						
	Age at SCT		1.04	1.00–1.08	0.078						

ALL: acute lymphoid leukemia, AML: acute myeloid leukemia, cy: cyclophosphamide, flu: fludarabine, Gy: Gray, MDS: myelodysplastic syndrome, RR: relative risk, SCT: stem cell transplantation, TBI: total body irradiation.

**Table 4:** Overview of patient cohorts undergoing TBI with a long-term follow-up. Analyses were only included if toxicities of 2 or more organs were reported. Studies with selective approaches based on IMRT were not included. 2nd mal.: secondary malignancies; card.: cardiac; FU: follow-up; fx: fraction; Gy: Gray; M: months; n.a.: data not provided; neuro.: neurological; pulmo.: pulmonary; TBI: total body irradiation; y: year.

Study	*n*	Age (y)	FU (M)	TBI Dose	Pulmo.	Card	Renal	Ocular	Neuro.	2nd Mal.
Belkacémi, 1998 [21]	326 (118 alloSCT)	3–63 (median 30)	68	10 Gy/1 fx 12 Gy/6 fx	19% *	n.a.	n.a.	cataract: 8% *	n.a.	n.a.
Bölling, 2011 [19]	120	18–70 (mean 46.1)	23	4–12 Gy/2–6 fx	20.4% (8.6%) **	n.a.	12.8%	cataract: 8.6%	n.a.	5.8%
De Felice, 2016 [22]	211 (48 adults)	3–53 (median 14)	40	12 Gy/6 fx	9% (adults: 8.3%)	n.a.	n.a.	cataract: 12.8% (adults: 10.4%)	n.a.	0%
Marnitz, 2014 [20]	110 (62 alloSCT)	17–54 (mean 34)	68	n.a.	15.5% *	n.a.	n.a.	28% *	n.a.	0%
Thomas, 2001 [18]	186 (42% alloSCT)	15–60 (median 36.5)	55	10 Gy/1 fx 12–13.5 Gy/6 fx	19% *	n.a.	n.a.	28% * (15% cataract)	n.a.	n.a.
Present study	322	18–74 (median 47)	68	8–12 Gy/4–6 fx	23.9%	14.0%	20.2%	23.6% (6.2% cataract)	23.9%	5.3%

* Percentages are given for the total cohort (including autologous SCT). ** In this analysis, 8.6% designates pulmonary events without recurrent infectious events.

## Data Availability

Data relevant to this study are presented in the paper. The public deposition of data is not possible due to restrictions put in place by the Institutional Review Board.
